# Editorial: One Health in clinical microbiology

**DOI:** 10.3389/fcimb.2024.1404276

**Published:** 2024-04-15

**Authors:** Leshan Xiu, Kokouvi Kassegne, Jianhai Yin

**Affiliations:** ^1^ School of Global Health, Chinese Center for Tropical Diseases Research, Shanghai Jiao Tong University School of Medicine, Shanghai, China; ^2^ One Health Center, Shanghai Jiao Tong University-The University of Edinburgh, Shanghai, China; ^3^ National Institute of Parasitic Diseases, Chinese Center for Disease Control and Prevention (Chinese Center for Tropical Diseases Research), NHC Key Laboratory of Parasite and Vector Biology, WHO Collaborating Center for Tropical Diseases, National Center for International Research on Tropical Diseases, Shanghai, China

**Keywords:** One Health, clinical microbiology, human, animal, environment

With the understanding that the health of humans, animals, and the environment are intricately connected, the One Health approach has emerged as a powerful framework for addressing complex health challenges (Mwatondo et al., 2023). This approach emphasizes collaboration across sectors, disciplines, and communities to foster well-being and mitigate threats to health and ecosystems (Chen et al., 2022; Nyokabi et al., 2023). In the context of clinical microbiology, where the dynamics of infectious diseases are shaped by interactions between pathogens, hosts, and the environment, the principles of One Health are particularly relevant.

In this Research Topic, titled “*One Health in clinical microbiology*,” we have gathered a collection of five manuscripts that embody the principles of One Health. These manuscripts offer diverse challenges and perspectives, and present recent advances in adopting a One Health approach to combat infectious diseases. Through investigations spanning clinical characteristics, epidemiology, molecular characterization, and case reports, this Research Topic highlights the importance of cross-sectoral, interdisciplinary, and community collaboration to promote well-being and mitigate threats to health and ecosystems.

As global populations recover from the recent COVID-19 pandemic, attention has turned to the origin of the disease and the multidirectional relationships among animal health, environmental factors, and human health (Chen et al., 2022; Keusch et al., 2022). Microorganisms play a crucial role in the One Health paradigm, serving as key connectors among human, animal, and environmental health. While previous One Health research has predominantly focused on microbial pathogens responsible for zoonotic diseases (Nyokabi et al., 2023), attention is now turning to non-tuberculous mycobacteria (NTM) strains. These resilient pathogens, which are resistant to standard anti-tuberculosis drugs, present therapeutic challenges and are ubiquitous in water, soil, and dust. The study “*Clinical Characteristics of Patients with Non-Tuberculous Mycobacterial Pulmonary Disease: A Seven-Year Follow-up Study Conducted in a Tertiary Hospital in Beijing*” by Liu et al. reported the clinical manifestations and outcomes of non-tuberculous mycobacterial pulmonary disease. These findings emphasize the need to understand the zoonotic potential and environmental reservoirs of mycobacteria, highlighting the importance of the One Health strategy in preventing and controlling zoonotic tuberculosis.

In the clinical setting, One Health provides practical ways to incorporate environmental contact considerations into patient care. Despite endorsements from major medical and public health organizations, studies have revealed limited awareness among physicians regarding the environmental health aspects of medicine. Understanding the clinical characteristics and risk factors for bloodstream infections in patients with solid tumors is crucial for effective management. Another study, “*Clinical characteristics of bloodstream infections in adult patients with solid tumors and a nomogram for mortality prediction: a 5-year case-controlled retrospective study in a tertiary-level hospital*” by Xue et al. provided valuable insights into bloodstream infections in immunocompromised patients, highlighting the need for integrated approaches to infection prevention and control in healthcare settings.

Traditionally, One Health research has focused primarily on zoonotic microbial pathogens. However, it is essential to recognize that animal diseases can directly stem from pathogen sources in soil (Jourdan et al., 2018), with potential transmission to humans. Indigenous populations worldwide face disproportionately high rates of diseases related to their living environment and animals. Brazilian indigenous communities are particularly vulnerable to toxocariasis, with associated risk factors including poor infrastructure and contact with contaminated river water. Transmission may also occur through waterborne routes, with embryonated eggs likely spreading to water supplies by rain. Expanding beyond traditional clinical settings, a study in our collection explored the One Health approach to fight toxocariasis in Brazilian indigenous populations (Alvares Santarém et al.), emphasizing the intricate links among human health, animal reservoirs, and environmental contamination.

Globalization and intensive farming practices are amplifying the risks associated with foodborne diseases, facilitating the spread and mutation of pathogens. Simultaneously, these practices foster environments conducive to disease transmission and the emergence of antimicrobial resistance. The study “*Molecular characterization of Listeria monocytogenes strains isolated from imported food in China from 14 countries/regions, 2003-2018*” provided critical evidence on the genetic diversity and epidemiology of *Listeria monocytogenes* in imported food products (Zhu et al.), illustrating the importance of surveillance and regulation in mitigating the risk of cross-border foodborne illness.

Recent advances in omics and statistical approaches have highlighted the importance of technological innovations in understanding and addressing health challenges. Indeed, techniques such as metagenomic next-generation sequencing (Muloi et al., 2023), offer promising avenues for enhancing our diagnostic capabilities for zoonotic infections. These advances underscore the pivotal role of technology in advancing One Health initiatives, as demonstrated by a manuscript presenting a case report of the diagnosis of cat-scratch disease using metagenomic next-generation sequencing (Zhou et al.).

In conclusion, these studies in the current Research Topic underscore the importance of adopting a One Health approach in clinical microbiology research and practice, covering a diverse range of topics, providing valuable insights into the interface between human health, animal health, and the environment, helping to advance One Health principles in clinical microbiology, and stimulating further interdisciplinary collaborations in the pursuit of global health security and sustainability. As highlighted in these manuscripts, urgent action is warranted, particularly in addressing zoonotic diseases, ensuring food security, and managing clinical microbiology-related health emergencies. Looking ahead, it is essential to recognize that the One Health concept is dynamic and evolving. As new interfaces between disciplines are discovered and accessible, the One Health approach will continue to encompass more disciplines. The real challenge moving forward lies in better understanding the interfaces between human, animal, and environmental health and addressing health challenges at the system level. By embracing the dynamic nature of the One Health concept and leveraging knowledge across disciplines, we can better anticipate and respond to emerging health threats, ultimately contributing to the well-being of populations and ecosystems.

## Author contributions

LX: Writing – review & editing, Writing – original draft, Visualization, Supervision, Formal Analysis, Conceptualization. KK: Writing – review & editing, Visualization, Formal Analysis. JY: Writing – review & editing, Visualization, Formal Analysis.
